# Lifting the cage of consent a data perspective on evolvable trust relationships

**DOI:** 10.1016/j.dib.2026.113216

**Published:** 2026-09-17

**Authors:** Beatriz Esteves, Ruben Verborgh

**Affiliations:** IDLab, Department of Electronics and Information Systems, Ghent University, Belgium AA Tower / Ghent University – imec, Technologiepark-Zwijnaarde 122, 9052, Ghent, Belgium

**Keywords:** GDPR, Technolegal, Systems

## Abstract

Those concerned about privacy worry that personal data changes hands too easily. We argue that the actual challenge is the exact opposite: our data does not flow well enough, cultivating a reliance on questionable and often unlawful shortcuts in a desperate bid to survive within today’s data-driven economy. Exclusively punitive interpretations of protective legislation equally hinder “doing the right thing” and “doing the wrong thing”, in an abject mistranslation of how ethical choices correspond to financial cost. As long as privacy-friendly data treatment proves more expensive or complicated than readily available alternatives, economic imperatives will continue to outrank their legal counterparts. We examined existing international and European legislation, looking at both requirements for consent and other legal grounds that go beyond it, with the aim of facilitating mutually beneficial interactions, rather than more narrowly focusing on the prevention of undesired behaviors. Building on this legal reasoning, we propose the implementation of evolvable trust systems as a scalable alternative to the omnipresent yet deeply broken delusion of ill-informed consent. Our proposal encourages a redirection of additional efforts towards the technolegal alignment of economic incentives with societal ones, reminding us that—while trust remains an inherently human concept—technology can support people in evolving and scaling their relationships to meet the increasingly complex demands of current and future data landscapes.

Specifications Table

**Table:** 

Subject	
Specific subject area	*Special issue European Data Spaces*
Type of data	*n/a*
Data collection	*n/a*
Data source location	*n/a*
Data accessibility	*n/a*
Related research article	*n/a*

## 1 Introduction

### 1.1 The limitations of privacy-centric framings in public discourse

Public discourse concerning personal data has been influenced, to a considerable extent, by periodic reports of large-scale data misuse involving major platform operators. Widespread reporting on incidents such as Cambridge Analytica [1] and a number of subsequent events [[2], [3], [4], [5]] has taken place within broader economic and political conditions that may themselves have contributed to the emergence of such practices. Certain journalistic accounts tend to present privacy as a unidimensional variable to be maximized, an approach which may, in some cases, be aligned with the communicative interests of the data-processing entities that are the subject of such reporting. Following public statements regarding the diminished contemporary relevance of privacy [6], the public communications of major technology companies have increasingly incorporated privacy-related framings [[7], [8], [9], [10]]. Such framings have, in turn, contributed to sustained societal engagement with the concept of privacy, though the resulting discussions may involve a degree of terminological broadening that complicates precise analytical use of the term. In parallel, commercial concerns regarding limitations on data availability and individual concerns regarding potential misuse of personal data appear to co-exist and to be mutually reinforced by competitive pressures encouraging the acquisition of personal data across a wide range of contexts.

Corporate communications emphasizing privacy do not, in themselves, address the full range of considerations relevant to personal data governance, given that maximal privacy, although conceptually well-defined, is associated with a limited set of practical use cases when considered in isolation. This may be illustrated by means of a hypothetical scenario in which an individual undertakes the physical destruction of all copies of their personal records and associated electronic devices, and further exercises the right to erasure provided under the General Data Protection Regulation with respect to all remaining controllers. Such an individual would attain a state approximating complete privacy over the personal data in question, while concurrently forgoing practical access to the same data for their own purposes. The preceding illustration, although stylized, points to a more general observation, namely that analytical frameworks constructed exclusively around the concept of privacy may not adequately capture the broader set of protection-related requirements that arise in practice for data subjects. Privacy is understood in the present work as a multidimensional construct, and no attempt is made herein to reduce it to a single analytical dimension. For the specific purpose of examining decisions relating to data exchange, however, we refer to the *CIA triad* [11], depicted in Fig. 1, which frames such decisions in terms of an ongoing set of trade-offs among the properties of Confidentiality, Integrity, and Availability.

**Fig. 1:**
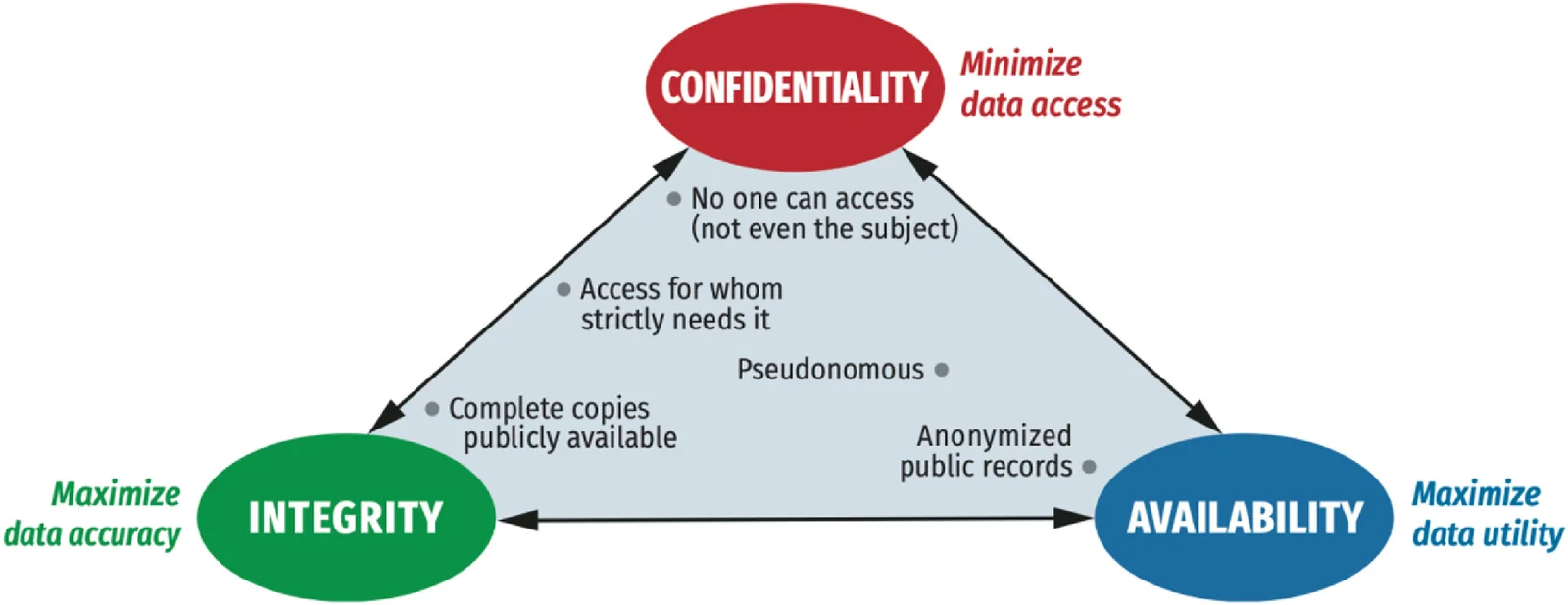
The CIA triad [11] of Confidentiality, Integrity, and Availability illustrates that personal data requirements involve nuance and complexity beyond what any one-dimensional privacy lever can model. One can never maximize all three; the appropriate degree of compromise is a case-specific choice.

The subsequent analysis is oriented primarily towards the confidentiality dimension, while acknowledging that privacy, understood as a broader construct, additionally encompasses considerations pertaining to integrity, availability, and contextual norms governing information flow. A degree of tension may be observed between the confidentiality and availability dimensions: mechanisms such as data redundancy and backup provisioning, while contributing to improvements in the reliability of data access, concurrently give rise to an increase in the number of potential pathways through which unauthorized disclosure of the data in question may occur. More generally, modifications to the accessibility of a given data resource tend to exert comparable effects on both unauthorized and authorized forms of access. This phenomenon may be observed in a range of access-control contexts in which measures intended to restrict access by unauthorized parties concurrently introduce friction for authorized parties under certain operational conditions.

A legislative framework designed to provide support to natural persons in relation to their day-to-day activities and longer-term objectives is accordingly required to take into consideration the circumstances under which, and the modalities by which, personal data may contribute to the attainment of such objectives. It cannot be assumed that the maximization of confidentiality constitutes a universally optimal configuration across the full range of data-exchange relationships that may arise in practice; the remaining portions of the associated parameter space are also to be characterized if legal and technological instruments are to deliver substantive forms of empowerment to the data subjects concerned. In the absence of such characterization, regulatory developments may be reduced, in practical terms, to the mitigation of risk, rather than to the facilitation of the mutually beneficial forms of data exchange that data protection frameworks are, in principle, intended to support.

### 1.2 Personal data as an input to bilateral value exchange

A more nuanced discussion requires shifting our perspective from personal data as solely a risk or liability, to personal data as an intentional means for extracting desired and mutual value from business-to-customer and other relationships. For the purposes of the following analysis, a number of subtleties and complexities are provisionally abstracted away in favor of a simplified interaction model involving a single individual and a single service provider, so as to permit a focused examination of the underlying motivations and incentive structures associated with such exchanges.

Whenever an individual requests a certain service from a third-party provider, the nature of this service may inherently depend on the availability of *i)* relevant data points *ii)* for the purpose of executing the service at the requested quality level *iii)* during the period of its execution. From an efficiency and confidentiality perspective, we can reasonably expect the individual to prefer *data minimization* [12]—in absence of accidental or deliberate barriers that distort the exposure of additional data into the more simple or attractive option [13,14]. Minimization involves limiting the necessary exchange to *i)* those data points strictly required for any proper execution of *ii)* only the specifically requested service within *iii)* solely the time and space required to execute the service. In terms of the CIA triad, successful minimization thus trades the *smallest possible decrease in confidentiality* for the *smallest possible increase in availability* to complete the requested service, with *possible compromises on integrity* for convenience.

For the purpose of illustrating a strict application of the model described above, consideration is given to the case of a printed magazine subscription. This service may be characterized as a transaction in which a monetary consideration is provided by the customer in exchange for the postal delivery, on a monthly basis over a period of twelve months, of a specifically designated magazine publication. The initial payment procedure is considered to fall outside the scope of the present analysis, which is oriented towards the delivery component of the transaction. Impacts on the confidentiality dimension are minimized in the scenario in which the magazine publisher retains only the postal address of the customer, the identification of the selected publication, and the termination date of the subscription in question, with all such data items being subject to secure deletion following the delivery of the final issue. From a theoretical standpoint, the aforementioned data items may be regarded as sufficient to enable service providers to deliver the subscription service without recourse to additional data collection or processing activities. Each party to the transaction may be understood to act in a rational manner and in accordance with its own respective interests: the customer is understood to attribute higher utility to the receipt of the magazine than to the retention of the corresponding monetary amount and postal address, while the service provider is understood to hold the inverse preference ordering.

Notwithstanding the operational efficiency associated with the minimization approach outlined above, it should be noted that its application does not, in itself, serve to prevent or to mitigate the full range of privacy-related risks that may arise. Given that a transfer of personal information has taken place in the course of the transaction, it may be argued that some degree of impact on the customer's privacy has occurred, irrespective of any characterization of such impact as necessary for the purposes of service provision. Although neither the preservation nor the compromise of privacy constitutes a primary motivating factor for either party to the interaction, the combination of voluntary participation with data minimization does not, on its own, provide a sufficient safeguard against subsequent misuse, including in relatively straightforward scenarios. The minimal set of data items considered in the present example may, for instance, be regarded as sufficient to enable third parties with unlawful intent to identify potential targets for property-related offences on the basis of subscription patterns associated with publications addressing specific subject areas, in combination with residential location data. In contrast to a more narrowly construed one-dimensional privacy axis, the broader analytical framework provided by the CIA triad accommodates the exercise, on the part of each individual customer, of a right to determine whether their continued interest in a given subject area is considered to justify a marginal elevation in the associated level of exposure to property-related risk. The correct application of the minimization principle nevertheless contributes to a reduction in the overall risk level, by limiting the attack surface associated with the transaction to the minimum extent compatible with the delivery of the requested service to those parties electing to obtain it.

In practice, the scope of data to which service providers seek access tends to be larger—sometimes considerably—than the strictly minimal scope required to deliver a requested service, implying that other motives are at play. Financial incentives might drive them to bypass data minimization with extensions of *i)* collected data points, *ii)* purpose, and/or *iii)* time and space. Perhaps the provider operates a different business model, in which their primary source of revenue is not subscriptions but third-party services based on extended subscriber data. An extension of purpose occurs when the provider retargets subscription data for different objectives, such as marketing for its other periodicals. Providers commonly retain customer data past the end date of their subscriptions, even if only as a service to themselves for advertising or speeding up future subscriptions. Such nonessential extensions expand the attack surface or prolong its exposure, leading to a heightened risk of various kinds of misuse—and the provider’s own violation of minimality already constitutes a first instance thereof.

From the simple exchange model, two broad modi operandi emerge:

1. Service providers for whom the execution of directly requested services is the exclusive source of long-term income. (Incorrectly) assuming an impeccable inflow of customer data, such providers lack financial incentives to process any data beyond the scope of each service’s execution. This first category counts a limited number of members today, as few companies restrict the storage of customer data to the scope of explicitly requested services.
2. Service providers who supplement their long-term gains by repurposing data gathered in the context of one service for the realization of other services, offered to either themselves or different classes of customers. Most companies reside in this second category, as their business model is predicated on their ability to leverage data for additional services. Given the high cost of data acquisition at any point in time, they opt for an extended scope in advance, even though excessive in the moment.

This chasm could be closed with complementary strategies. A punitive approach considers what barriers and obstacles could be imposed onto the second group to nudge or force a substantial portion of them into behaving like the first, which ideally remains unaffected by the intervention. A supporting approach instead asks which barriers and obstacles could be removed for the first group in order to encourage voluntary movement by the second, increasing the former’s attractiveness for all parties involved.

### 1.3 Frictions in data exchange and associated socioeconomic costs

The theoretical treatment of intentional data exchange developed in the preceding sections requires further extension in order to incorporate considerations of an ethical and legal nature. Without prejudice to any normative evaluation of specific commercial models, it may be observed, on a descriptive basis, that (a) a given individual may seek to obtain a service from a service provider, (b) the exchange of certain categories of personal data may constitute a precondition for the satisfactory provision of the service in question, and (c) the service provider may, independently of the requirements of the service, possess an incentive to undertake processing of personal data beyond the scope of such requirements. For the purposes of the subsequent analysis, the position is adopted that non-minimal patterns of data usage give rise to negative societal effects over the longer term, a proposition for which a degree of empirical support has been documented in the literature of recent years. On the basis of the foregoing, it falls to the legislator to ensure that the longer-term benefits accruing to service providers from the application of data minimization are of sufficient magnitude to outweigh any short-term or longer-term competitive advantages that would otherwise result from the non-application of that principle. Legislative instruments may achieve this outcome by reducing the benefits associated with undesired forms of conduct and/or by increasing the benefits associated with desired forms of conduct.

The *General Data Protection Regulation (GDPR)* [15] may be understood, in part, as a regulatory response to the observed proliferation of data-processing practices considered to be unsustainable in the longer term, which the Regulation seeks to disincentivize by means of enforcement procedures that may give rise to administrative fines of considerable magnitude [16,17]. Entities engaged in the processing of personal data are required to maintain the operational capacity necessary for the execution of certain legally prescribed procedures, the outputs of which may subsequently serve as evidence of either compliance or non-compliance with the applicable requirements. The establishment and maintenance of such capacity is associated with non-negligible costs in both cases, a circumstance which may, in certain configurations, give rise to an outcome in which entities possessing sufficient financial resources to absorb the fines associated with non-compliance elect, on the basis of an ex-ante cost assessment, to treat such fines as a recurring category of operating expenditure. Under this enforcement-oriented interpretation, the effect of the GDPR in relation to its objective of establishing a level playing field may be characterized as one in which individuals and the majority of undertakings are positioned in a broadly comparable relation of limited leverage vis-à-vis multinational entities, for which the maximum administrative fine of 4% of annual global turnover may be assimilated to a category of foreseeable operational expenditure.

Notwithstanding any normative considerations, the interpretation described above constitutes a rational inference from the manner in which the GDPR operationalizes the relationship between longer-term societal costs and legal consequences by means of a short-term economic instrument. Commercial undertakings act in accordance with the principle of rational agency and are subject to fiduciary obligations vis-à-vis their shareholders which require conduct that is financially predictable and consistent with prevailing standards of financial responsibility. The Republic of Ireland, which serves as the location of the European headquarters of a substantial number of global data-processing undertakings, has expressed reservations with respect to enforcement approaches perceived as restrictive of jurisdictional competitiveness. The Irish government has, in this context, adopted a regulatory posture oriented towards positive-incentive instruments with respect to commercial conduct in the data-processing sector, in preference to constraint-based instruments [18]. The objective of the European legislator with respect to the deployment of proportionate enforcement measures as a means of achieving alignment, over the longer term, between societal and economic incentive structures may be considered to have been affected, in part, by sustained advocacy activities undertaken on behalf of stakeholder groups whose stated preferences are oriented towards a divergence between such incentive structures [19]. The externalization of privacy-related costs may, in certain configurations, be achieved through formalized institutional mechanisms without recourse to explicit public statements regarding the status of privacy as a societal value.

A further consequence which may be regarded as unintended is that the same legislative framework may, in practice, act as a disincentive to data-exchange arrangements characterized by the pursuit of mutual benefit by all participating parties in the absence of undisclosed forms of data exploitation. Any remaining incentive for an undertaking to adopt a data-minimizing operational model tends to be attenuated by the elevated cost and risk profile associated with the processing of personal data in general. Undertakings may, on the basis of a comparative cost assessment, conclude that the avoidance of data minimization constitutes a more efficient course of action, given that the marginal difference in operational effort between minimal and non-minimal data-processing configurations is, in many cases, of limited magnitude relative to the fixed cost threshold associated with data acquisition activities that is common to both configurations. Without prejudice to those provisions of the GDPR that are oriented towards the empowerment of data subjects, including the provisions relating to the withdrawal of consent and to the right to erasure, the continued prevalence of data practices with negative societal implications may be considered to have implications for the position of the European Union with respect to international data governance [20]. Comparable effects may be observed in the more recent *AI Act* [21], the design of which reflects an attempt to reconcile protective and innovation-oriented objectives, and in a number of legislative instruments adopted or under consideration outside the European Union, including the proposed *Brazil AI Bill* [22], India’s *Digital Personal Data Protection Act* [23], and Tanzania’s *Personal Data Protection Act* [24].

In light of the limitations identified above in relation to existing protective mechanisms, an initial reading might suggest that personal data circulates with an insufficient degree of friction. The present analysis, however, arrives at the opposing conclusion. Personal data may be characterized as circulating with insufficient effectiveness, a state of affairs which may account for the observed prevalence of alternative practices, within which the ex-ante incorporation of potential administrative fines into the overall cost basis of data acquisition activities is identified, on the basis of a comparative cost assessment, as the more efficient and, accordingly, the more rational course of action. Such patterns of conduct may be regarded not as unforeseeable outcomes, but rather as observable consequences of limitations in the existing legislative framework, arising within an economic context in which the operational capacity required to accommodate current developments in the fields of data processing and artificial intelligence is subject to material constraints.

The suboptimal functioning of personal data flows may be considered to give rise to societal consequences that extend beyond reductions in commercial operational efficiency. A number of impediments to data circulation, which may present, on a preliminary assessment conducted from a confidentiality-oriented perspective, as consistent with protective objectives, may in certain configurations be considered to operate as constraints on the ability of data subjects to exercise informed choice in relation to trade-offs involving a limited reduction in confidentiality in exchange for a substantive increase in availability. The consequences associated with the immobilization of personal data may be of considerable significance for the affected data subjects. The timely transmission of data generated by wearable fitness-monitoring devices to appropriately qualified medical personnel may be a determinant of patient outcomes [25]. Data generated in the course of the operation of motor vehicles may contain indicators consistent with the presence of Alzheimer's disease in the vehicle operator, preceding clinical identification by a medical practitioner [26]. Taken together, the illustrations provided above indicate that any proposed approach to the enhancement of data-flow effectiveness would be required to take into account sector-specific operational requirements, applicable constraints, and associated risk profiles, which may be understood to shape the appropriate configuration of trust arrangements, governance frameworks, and technological safeguards in a given operational context.

In view of the foregoing, further consideration is warranted of the potential improvements that may result from a revised approach to personal data flows, including improvements that may offset the associated adverse effects. The illustrations provided above support the position that addressing the observed scarcity of well-functioning data flows may contribute to a reduction in the incidence of harmful data flows. A balanced approach to data access serves the interests of both the data subject and the entities responsible for processing personal data in the course of service provision. The analysis of mutual benefit may assist in the identification of favorable configurations within the CIA triad. An operational legal framework is nevertheless required in order to translate the preceding theoretical considerations into a functional data-exchange framework.

### 1.4 The role of trust in longitudinal data relationships

In this article, we introduce a technolegal proposal for *evolvable trust* as a sustainable mechanism for data exchange with well-aligned socioeconomic incentives. We argue the need for a more long-term view on the evolving relationship between a data subject and a data controller. A relational approach can only succeed if it recognizes the needs and desires of all sides as valid and important: individuals have clear requirements in society beyond confidentiality, and companies have the reasonable goal of guarding their profit/investment ratio to maintain a competitive economic position. The ethical spectrum between those positions allows us to demarcate areas where those different demands are not necessarily in conflict, and to address the obstacles that obstruct such alignment.

On *both* sides of the relationship, trust is at low levels [27]. The frequently expressed proposition that data subjects do not accord trust to commercial undertakings with respect to the processing of their personal data may itself be regarded as one element of the broader projection dynamic that characterizes contemporary discourse on privacy. The distrust expressed by data subjects may be understood, in part, as a reciprocal response to the distrust extended towards them by commercial undertakings. The prominent consent dialogs presented at the initial stage of most online interactions may be interpreted as an expression of the position, on the part of the undertaking, that the data subject cannot be assumed to grant, at a subsequent point in time, agreement to an anticipated requirement for the processing of personal data. The depth of this position, in combination with the limited incentives associated with the application of data minimization, contributes to the establishment of an interaction design of considerable complexity, the effect of which is to constrain the ability of the data subject to exercise a preference for data minimization. The result may be characterized as a self-reinforcing dynamic, in which the assumption, on the part of commercial undertakings, that data subjects will not accord them trust, gives rise to communications directed at data subjects the content of which reflects the same assumption.

From a legal perspective, commercial undertakings may be considered to make use of asymmetries in bargaining position to transfer a substantial portion of their compliance obligations to data subjects, on the basis of the underlying assumption that deficits in trust may be addressed on a unilateral basis through the provision of contractual or informational text. This tendency may be considered in the context of the differing scalability characteristics associated with distinct categories of bilateral relationship. Business-to-business interactions are, in aggregate, of an order of magnitude lower than business-to-consumer interactions in terms of volume. Undertakings engaged in business-to-business interactions are, accordingly, more frequently in a position to allocate the time and operational capacity required to negotiate legal contracts governing the terms of such interactions. Developments in the field of machine-assisted contract drafting may be understood to further support the scalability of this approach [28]. In contrast, the prevailing reliance on consent [29,30] as a legal basis for data exchange, which does not lend itself to automation [31], gives rise to scalability limitations of a magnitude that may be considered to approach the point of practical infeasibility. Websites presenting a volume of privacy notices that exceeds the capacity of the data subject for review may be regarded as contributing to the aggregate costs associated with the drafting of such documentation and with the corresponding review activities on the part of the data subject [32]. The pattern of interaction described has been characterized in the literature as "the biggest lie on the Internet" [33], a formulation which refers to the routine indication, by data subjects, of agreement to terms that have not been the subject of substantive review. Fig. 2 provides an illustration of this pattern.

**Fig. 2:**
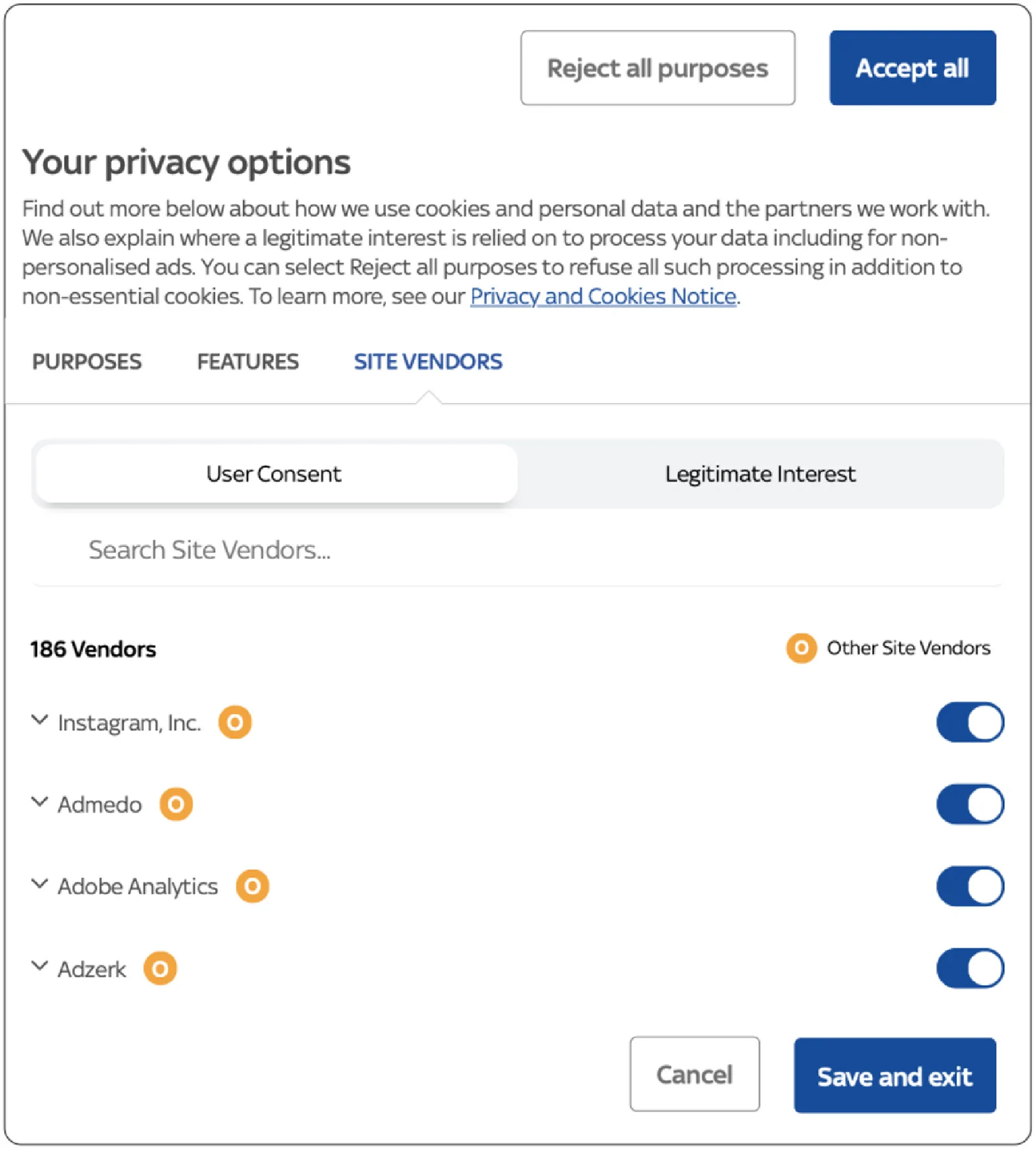
Cookie dialogs are up-front agreements of mutual distrust. The above specimen (courtesy of Sky News) presents one of the more recent fads where, in a most creative interpretation of the GDPR, visitors can seemingly “consent” to the use of legitimate interest as a legal basis for data processing.

Independently of ethical positions held by individual citizens, the core dynamic underlying the data-driven economy may be reduced, in analytical terms, to a question of economic organization. Considerations of an ethical nature, when examined over the longer term, converge on the problem of the construction of a sustainable equilibrium among the various monetary and non-monetary costs borne by the participating parties. The current configuration may be characterized as a mutually disadvantageous arrangement between data subjects and commercial undertakings, the parameters of which have, for some time, exceeded the conditions required for the maintenance of sustainability and, a fortiori, for growth. This configuration may nevertheless be considered as presenting an opportunity for reconfiguration: constraints on growth may, over time, come to represent material limitations on commercial activity. An analytical approach that assigns equivalent weight to all parties to the data-exchange relationship may contribute to the establishment of conditions under which certain participants may consider a departure from the current local optimum. The question may accordingly be formulated of whether a focus on mutual value could permit the identification of configurations that are advantageous to both parties to the relationship.

The present article examines the relationship between the objectives of the legal framework with respect to the codification of ethical data principles and the limitations of the same framework with respect to the prescription of such principles. Existing punitive responses to transgressive behaviors are complemented by our proposal for an unburdening empowerment that fosters mutually beneficial exchanges in a technology-assisted, scalable way. Two-sided cost reduction could spearhead the realignment of societally advantageous data minimization with economically motivated necessity. We ultimately target the long-term goal of making responsible data behaviors cheaper than their currently less expensive, unethical shortcuts. 2.1, 2.2 explore, first, the extent to which contemporary international legal frameworks remain dependent on consent as a primary legitimizing mechanism for personal data processing, and second, the function and evolution of consent within the broader landscape of European Union digital legislation developed under the European Data Strategy. In Section 3, we extend this analysis to the remaining legal bases for personal data processing under the GDPR, comparing them to consent in terms of their potential for automation, the degree of interaction they require from data subjects, and their capacity to scale within increasingly complex data ecosystems. Section 4 then articulates the concept of “trust” as the analytical foundation of this article, examining in particular its capacity to evolve as a key enabler of sustainable data and service exchanges between individuals and companies. Section 4.4 subsequently turns to the technical mechanisms capable of operationalizing this vision in practice. Section 5 concludes the article.

### 1.5 Objectives

Different scholars across different decades reached similar conclusions about the *trust* conundrum, with the difference that the legal and technical landscape that might finally allow for meaningful change. Never mind that, in the era of data spaces, we also need to piece together the legal jigsaw that data protection and AI compliance is becoming, especially in Europe. This conflicting nature is even present at the most basic level – safeguarding the fundamental right to health can come at the cost of the fundamental right to privacy. All in all, policy making and technology development have walked astray for far too long.

As such, in this article, we *i)* address challenges related to valid GDPR consent, *ii)* for scalable relationships between humans, businesses, and machines, and *iii)* explore other legal bases, towards establishing *long term* evolvable trust in the exchange of data, services, and goods. Considerations on fundamental facets of trust are explored in the hopes of establishing a methodology for the realization of such demands through a *permanently evolving* relationship. This approach leverages aspects of *i)* data governance, *ii)* automation, *iii)* interoperability, and *iv)* stakeholder collaboration, features previously identified as core for successful RegTech systems [34]. We further elaborate on these four foundational aspects as mutually reinforcing dimensions of evolvable trust. First, data governance provides the normative backbone upon which trust can be constructed and assessed. By defining clear rules, accountability mechanisms, and transparency obligations, it transforms trust from a subjective expectation into a verifiable condition, enabling participants to rely on shared standards rather than unilateral assurances. Second, automation functions as the primary scaling mechanism for trust. The automated verification of compliance and execution of data-sharing agreements reduces reliance on manual intervention, minimizes error, and allows trust relationships to adapt continuously in response to changing conditions and data flows. Third, interoperability operates as the technical conduit through which trust can persist across heterogeneous systems. By preserving the integrity of the underlying intent – such as describing verifiable conditions for usage – it ensures that trust is neither diluted nor lost when data traverses organizational and technological boundaries. Finally, stakeholder collaboration constitutes the social substrate of trust. By aligning legal, technical, and economic incentives among the actors involved, it supports a co-evolutionary dynamic in which trust is not statically imposed but progressively negotiated and refined. Taken together, these aspects illustrate how evolvable trust emerges from the interaction between normative structures, technical capabilities, and social alignment.

We identify core areas of technolegal work where these features will be prominent, namely on i) compliance and provenance checking, ii) reusable policies, iii) delegation of control, iv) negotiation on access and usage, v) personalized dialogs, vi) secondary data reuse, and vii) auditing. Fig. 3 provides a mapping between the identified RegTech dimensions for evolvable trust and the abovementioned technolegal work areas. Compliance and provenance checking are closely anchored in the data governance facet, as it concerns the continuous verification that data processing activities adhere to applicable legal and contractual requirements, while maintaining traceable records of data origin and transformation. By embedding accountability and transparency into the lifecycle of data, it renders governance rules observable and enforceable in practice. Furthermore, the auditing component reinforces the data governance dimension by providing the mechanisms through which compliance, accountability, and performance can be systematically assessed, e.g., by third parties and authorities. Reusable policies emerge at the intersection of data governance and automation, translating normative rules and individual preferences into standardized, portable, and reusable formats. This dual role supports both the consistency of governance frameworks and their scalable enforcement across systems. Moreover, the delegation of control is rooted in the automation pillar, addressing how individuals and organizations can entrust decisions to technical agents under clearly defined constraints. In environments characterized by scale and complexity, such delegation enables trust to persist without constant human intervention, provided that automated actions remain aligned with the intent of the delegating party. In this context, negotiation on access and usage is of fundamental importance as it lies at the intersection of automation and stakeholder collaboration. It encompasses the mechanisms through which parties define and refine the conditions under which data may be accessed and used, combining automated support for efficiency with participatory processes that reflect the evolving preferences, risks, and incentives of the actors involved. Finally, personalized dialogs are primarily connected to stakeholder collaboration, as they shape the interaction layer between data subjects and data controllers. By enabling tailored, intelligible, and context-sensitive communication, they foster mutual understanding and more balanced participation in the formation of trust relationships. To foster further data sharing, secondary data reuse bridges data governance and stakeholder collaboration, as it requires not only adherence to predefined legal and ethical constraints but also continued alignment with the expectations and interests of the parties involved. Ensuring that data can be repurposed responsibly, thus, depends on both robust governance structures and sustained engagement between stakeholders. Across all these areas, the interoperability dimension plays a cross-cutting role by providing the technical means through which the other dimensions can be realized in practice. By enabling systems to exchange, interpret, and enforce shared representations of data provenance and usage, interoperability supplies the connective infrastructure that allows governance rules, automated processes, and collaborative interactions to function coherently across organizational and technolegal boundaries. Section 4.4 will highlight interoperable standards that can be used to sustain this dimension.

**Fig. 3:**
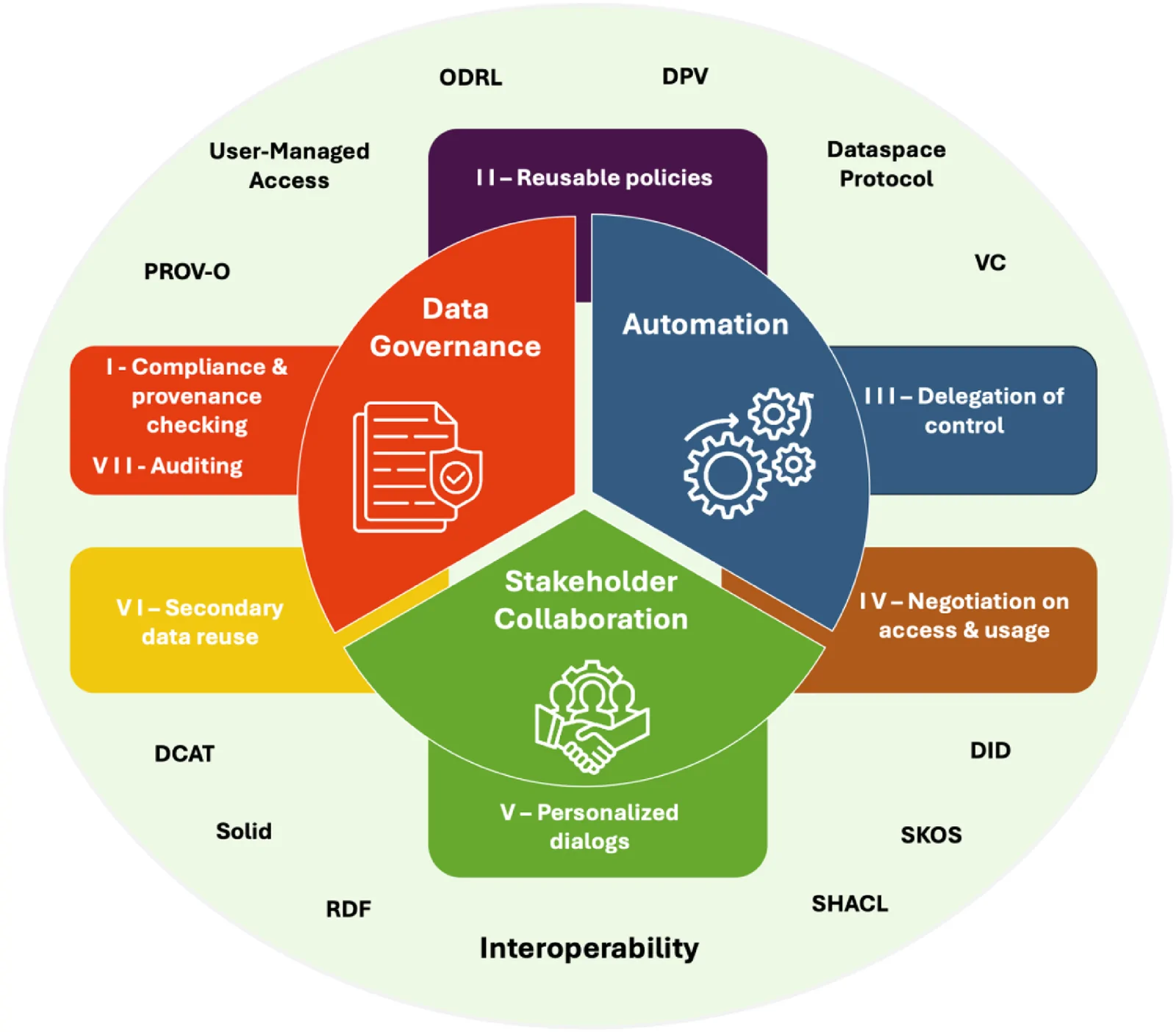
Mapping between identified RegTech dimensions for evolvable trust and technolegal work areas.

While we previously described foundational areas of work to implement the evolvable trust concept across data ecosystems, we must emphasize that the methodological scope of this contribution is intentionally foundational and exploratory. At this stage, our primary objective is not to provide empirical benchmarking or quantitative validation, but rather to articulate the structural and technolegal requirements that underpin the notion of evolvable trust. Accordingly, the analysis presented throughout this work relies on qualitative legal reasoning, i.e., 2, 3, as the foundational motivation for the establishment of evolvable trust, i.e., Section 4. This approach establishes the necessary theoretical grounding upon which future research can build, enabling subsequent validation through quantitative assessments and pilot implementations as the field and its supporting technologies continue to mature. Furthermore, the evolvable trust vision should be considered a generic, cross-sectoral framework that is applicable to any domain requiring long-term data exchange among distinct stakeholders.

## 2 Consent Cannot Scale

### 2.1 The universality of consent

Consent serves as the cornerstone for processing personal data on the Web [35], a principle upheld in both Europe and the United States, albeit through contrasting lenses. In Europe, the approach is an active one, requiring individuals to *opt-in*, with the intended goal of giving clear permission for their data’s use. Across the Atlantic in the US, the standard shifts, embracing *opting-out* as the norm, where consent is presumed unless it is objected to. Despite these divergent practices, both regions anchor their legality in the fundamental requirement that data subjects fully grasp the implications of their data’s journey and usage. Furthermore, a similar endeavor can be observed globally as new data protection laws emerge, inspired by GDPR’s influence. This wave of legislative activity is often described as the Brussels effect [36], where the influence of European Union standards ripples across the world, shaping data privacy frameworks far beyond its borders.

When we consider GDPR’s flavor of consent, its intent to empower the data subject shines brightly. However, it becomes equally apparent that it falls short for both individuals and businesses. What it brings in empowerment, it also brings in responsibility, placing the onus on data subjects to stay informed about every aspect of their data’s usage. This approach brings with it two significant challenges: *i)* a scalability burden, and *ii)* an understandability concern, as legalese can be overwhelming and obscure for individuals. Looking at its concrete conditions, consent should be provided through a *“clear affirmative action”*, be *“freely given, specific, informed and unambiguous”, demonstrable by the data controller*, and *“be as easy to withdraw as to give”* (Articles 4.11 and 7 [15]). These demands also manifest as scalability annoyances for businesses, particularly for small and medium enterprises, which might not have enough resources to scale effectively when faced with the challenges of meeting auditing duties and fulfilling obligations related to consent withdrawal. Furthermore, when businesses are faced with the decision of selecting a legal ground for processing the data necessary for their operations, they inevitably rely on obtaining consent to collect as much data as possible during the initial interaction, driven by the concern that users may not return in the future. This underscores, once again, that establishing a trust framework to facilitate the responsible flow of data will yield mutual benefits for both individuals and businesses. Not only that, the over-reliance on consent mechanisms also leaves companies at a disadvantage by not fully realizing the potential of data due to consent-imposed constraints.

As such, numerous voices have been raised against the efficacy of the consent mechanism to genuinely enhance the digital lives of people and businesses [35]—the culture of consent on the Web has morphed into uninformed adhesion to terms of service [37,38]. Moreover, beyond placing an impossible-to-deal-with burden on individuals, consent still is not valid since consent management platforms and other similar services are not even legally compliant for them to give informed and specific consent [39,40]. Furthermore, as Sun et al. highlight, an examination of GDPR enforcement, from its enforcement date to June 26th, 2023, reveals a substantial body of over 3000 official enforcement documents, among which 134 documents reference Article 7 on “Conditions for Consent” [38]. Of those, 65 cases resulted in fines, with only 7 imposing penalties exceeding 1 million euros. This analysis highlights a troubling reality: even if not lawful, the misuse of consent often goes insufficiently challenged, and it is difficult to challenge effectively.

Venturing beyond the borders of the EU, and examining the data protection landscape in the world’s most populous nations, the universal reliance on consent becomes strikingly clear. This reveals an equally pressing need to transcend the limitations of consent, recognizing that it is but a starting point in safeguarding personal data. Table 1 encapsulates these findings, drawing attention to key insights derived from the analysis of data protection regulations from India (IN), China (CN), US state laws, Indonesia (ID), Pakistan (PK), Nigeria (NG), Brazil (BR), Bangladesh (BD), Russia (RU) and Mexico (MX), the ten most populated countries in 2023^1^^,^^2^. Of these, only Bangladesh stands without a data protection law, though its Cybersecurity Act 2023 [41] launched a first step towards legislating the lawful use of identity information. Meanwhile, Pakistan’s Personal Data Protection Bill (PDPB) [42] remains in draft form, yet to solidify into enforceable law. Following we present a list of the examined laws per country.

1. India (IN) – Digital Personal Data Protection Act 2023 (DPDP Act) [23]
2. China (CN) – Personal Information Protection Law 2021 (PIPL) [43]
3. United States (US) – There is no comprehensive national data protection law in the United States, although there are laws focused on specific data types, e.g., consumer health data and minors’ online data, which are excluded from this analysis. Existing state laws, which are already being enforced, are described below.
  a. California (CA) – California Consumer Privacy Act (CCPA) [44]
  b. California (CA) – California Privacy Rights Act (CPRA) [45]
  c. Colorado (CO) – Colorado Privacy Act (CPA) [46]
  d. Connecticut (CT) – Connecticut Data Privacy Act (CTDPA) [47]
  e. Nevada (NV) – Nevada Privacy of Information Collected on the Internet from Consumers Act (NPICICA) [48]
  f. Oregon (OR) – Oregon Consumer Privacy Act (OCPA) [49]
  g. Texas (TX) – Texas Data Privacy and Security Act (TDPSA) [50]
  h. Utah (UT) – Utah Consumer Privacy Act (UCPA) [51]
  i. Virginia (VA) – Virginia Consumer Data Protection Act (VCDPA) [52]
4. Indonesia (ID) – Personal Data Protection Law 2022 (PDP) [53]
5. Pakistan (PK) – Personal Data Protection Bill 2023 draft (PDPB) [42]
6. Nigeria (NG) – Nigeria Data Protection Act (NDPA) [54]
7. Brazil (BR) – General Data Protection Law (LGPD) [55]
8. Russia (RU) – Federal law No. 152 FZ of 27 July 2006, “On Personal Data” (the Data Protection Act or DPA) [56]
9. Mexico (MX) – Federal Law for the Protection of Personal Data Held by Private Parties (FLPPDHPP) [57]

**Table 1:** Analysis of consent definitions and key requirements in the ten most populated countries in 2023 (the ISO 3166 standard was used to refer to countries and their subdivisions) versus EU's GDPR. Due to the absence of a comprehensive national law, the currently enforceable US state laws were analyzed in its place. Most laws opt for a GDPR-like definition, as showcased by the first nine rows, which focus on the definition of consent. The ensuing rows (Opt-out, Notice, Withdrawal, Delegation and Records) highlight key requirements derived from the usage of consent as a legal basis. Since California has two state laws on data protection, fields identified with a X* represent requirements present in both. (X) marks laws where withdrawal of consent is only considered for situations where opt-in consent is required for the processing of sensitive data, as opt-out consent is the norm. Pakistan's law is represented in X as it is still a draft and, as such, subject to change. Russia's notice requirements, as identified by X^DS^, depend on the source of the personal data, if the data subject is the data source, then the notice should be available upon request, while other sources require the notice to be shown before processing. Furthermore, Mexico's data protection authority promotes the availability of different types of notices, X^3^, depending on the type of data being processed and on how it is notified.

				US														
Criteria	EU	IN	CN	CA	CO	CT	NV	OR	TX	UT	VA	ID	PK	NG	BR	BD	RU	MX
**Affirmative act**	X	X		X	X	X	X	X	X	X	X		X	X				
**Freely given**	X	X		X	X	X		X	X		X		X	X	X		X	
**Specific**	X	X		X	X	X		X	X		X		X	X			X	
**Informed**	X	X	X	X	X	X		X	X	X	X		X	X	X		X	
**Unambiguous**	X	X		X	X	X		X	X	X	X		X	X	X			
**Unconditional**		X																
**Voluntary**			X				X			X								X
**Explicit**			X															
**Conscious**																	X	
**Opt-out**				X*	X	X	X	X	X	X	X							X
**Notice**	X	X	X	X*	X	X	X	X	X	X	X	X	X	X	X		X^DS^	X^3^
**Withdrawal**	X	X	X	(X)	(X)	(X)	(X)	(X)				X	X	X	X		X	(X)
**Delegation**		X				X		X	X									
**Records**	X	X	X	X*				X				X	X	X	X		X	

As evidenced in Table 1, it is apparent that the “Brussels effect” is more than just a buzzword. The majority of the analyzed laws reflect a definition of consent closely aligned with that of the GDPR, emphasizing its *freely given, specific, informed* and *unambiguous* nature, as well as it being an *affirmative act* taken by the data subject. Additionally, if we introduce the notion of consent being both *unconditional* and *conscious*, it becomes clear that further reflection is required on how such expectations would strain already overburdened consent-based systems.

In this context, of interest is also the choice of US state laws and their Mexican counterpart, as they have chosen to adopt “opt-out” consent as the default approach, while reserving “opt-in” consent as the required legal basis for processing sensitive personal data, such as health or children’s data. However, it must be stated that both regulatory approaches grant individuals the right to withdraw consent, with the exception of Texas, Utah and Virginia. The same cannot be said about record-keeping. While “opt-in” based jurisdictions require consent to be demonstrable, their “opt-out” counterparts mostly do not, explicitly, identify such a requirement.

The duty to showcase an appropriate consent notice, enabling individuals to make informed decisions and allowing businesses to obtain high-quality data, is prevalent across countries, while only Mexico’s data protection authority offers guidelines for three distinct types of notices, tailored to the nature of the data being processed and the method of notice delivery [58], e.g., a comprehensive notice is required when data is collected in person, a simplified version is needed for direct online or phone interactions, while a short form notice can be delivered when space is limited, such as in a text message. On that note, Russia’s Data Protection Act [56] notice requirements are also distinct from all others: depending on whether the data is collected from the data subject or from other sources, the duty to provide notice differs — in the first case, an active approach must be taken by the data subject, as the notice must only be available upon request, while in the second case, the law imposes a stricter requirement as the data subject must be provided with the notice prior to the beginning of the personal data processing. This analysis does not delve into the specific contents of the notices, which vary across jurisdictions. While these notices serve as key tools for informing individuals when consent is required, their function extends beyond consent alone. Businesses utilize them to meet broader transparency obligations, covering various legal grounds for processing data, outlining how individuals can exercise their rights, and more. Therefore, the intricacies of notice content fall outside the primary focus of this section. It could however be debated whether European policy-makers could take a page from the other jurisdictions’ books and allow the usage of distinct types of notices with more or less information according to the usage of data being made, its sensitivity, the preferences of the data subject, etc. Such an approach could benefit both sides of the equation, sparing users from engaging with details they deliberately choose to avoid, while also encouraging businesses to steer clear of the shortcuts they currently employ to gain rapid access to data.

Another interesting idea is being put forward in India’s DPDP Act [23], as well as a few of the US state laws [47,49,50] — the idea of having a “consent manager”, or an “authorized agent”, acting on the data subject’s interest. While the role of US-based “authorized agents” is quite limited in terms of scope, i.e., they only act on the person’s behalf to opt-out of processing activities that do not align with the data subject’s preferences, India’s “consent manager” has a much bigger role to play. The DPDP Act actually defines that such a manager can act as “a single point of contact [...] to give, manage, review and withdraw [..] consent through an accessible, transparent and interoperable platform” [23]. Furthermore, US-based laws put forward techniques to designate such agents including browser settings or extensions, or a global setting embedded within an electronic device. The significance of such techniques cannot be overstated—*every* business-to-customer interaction inherently faces greater scalability challenges compared to business-to-business interactions since they are far more numerous, and most customers do not have a proficient capacity to engage in meaningful negotiations. Therefore, equipping individuals with robust tools that can negotiate on their behalf will allow them to manage their personal data far more effectively and efficiently. In Section 4.4, we revisit this concept, highlighting the significant role Web agents will play in delegating control over data management and enhancing user autonomy.

Regardless of opt-in or opt-out conditions, or even what information needs to be passed in notices or kept in records, one fact remains clear: consent-as-is has reached its local optimum, in particular when it comes to empowering users or making new streams of data available to businesses. In response to these challenges, a growing number of legal scholars have proposed innovative ways to get over such a peak. One prominent suggestion is the concept of broad consent for secondary purposes, not specified at the time of consenting, such as storage or research use [59,60]. While these ideas have been optimistically put in perspective, they also come with trade-offs, especially when dealing with stringent requirements surrounding sensitive personal data, like health-related data. The possibility to use “Pre-Formulated Declarations of Data Subject Consent” put forward either by data controllers [61] or by data subjects [31], that have agents negotiating or enforcing preferences on their behalf [62], is also being discussed as a potential solution, though both still require advanced protection tools to avoid unfair terms for both parties.

### 2.2 Everything, everywhere, all at once

Over the past decade, technological progress has been met with a flurry of policy-making and legislative activity, particularly across Europe. While the notion of data protection is far from novel — its roots tracing back to the 1981’s Council of Europe Convention 108+ [63] and the EU’s 1995 Data Protection Directive [64] — the advent of the GDPR has reshaped the global landscape. This landmark regulation addresses the pervasive use of digital personal data, with consent being put at center stage, as underscored in the analysis provided in the previous section. In the wake of GDPR’s global effect, inspiring data protection regulations worldwide [36], the European Commission unveiled its *strategy for Data* in 2020 [65]. This initiative seeks to weave a seamless flow of data across sectors within the EU, while steadfastly upholding European ideals of personal data protection, consumer protection legislation, and competition law. At its core lies a commitment to crafting fair, transparent, and practical rules for accessing and using data, all underpinned by mechanisms of trustworthy governance to ensure integrity and balance in the digital age. A cascade of regulatory proposals has since emerged—many now enacted—each crafted to refine and expand the landscape of personal (and non-personal) data governance. From the Data Governance Act (DGA) [66] to the Digital Services Act (DSA) [67], Digital Markets Act (DMA) [68], Data Act [69], European Digital Identity (EUDI) [70], and the European Health Data Space (EHDS) Regulation [71], these measures collectively form a robust framework for the digital age. Furthermore, the AI Act [21] framework also cannot be ignored, as it regulates the usage of personal and non-personal data to train AI models. The timeline of these regulations, from the proposal date to its entry into force and applicability, is showcased in Fig. 4. With many obligations already in motion—spanning the DMA’s rules for large online platforms, or “gatekeepers”, the DGA’s mandates for data intermediation service providers and data altruism organizations, and the DSA’s framework for digital service providers—the European strategy for data is advancing with unwavering momentum, with many already calling for reforms to the DGA [72,73]. Moreover, the recently adopted EHDS Regulation cannot be forgotten, as it imposes restrictions on the use of personal and non-personal electronic health data whether being for primary care usage or for promoting the secondary use of health data for research, innovation, and policymaking. Member States will be required to offer at least one digital identity wallet to all citizens and residents by 2026, as mandated by EUDI [70], and its Implementing Acts for the core functionalities and the certification of EUDI wallets, which were recently adopted by the Commission [74]. To further underscore the point, GDPR enforcement rules [75]—additional requirements to ensure the consistent interpretation and application of the GDPR across the EU—and GDPR simplification rules [76]—an amendment to reduce record-keeping for small and medium-sized enterprises—were also proposed by the Commission. Amid the still-unraveled intricacies of these regulations, and their relationship with the GDPR, one truth emerges—consent has been elevated to a central role within this evolving regulatory framework [77]. As such, in the following paragraphs, we will explore these regulations and the role that consent plays in them.

**Fig. 4:**
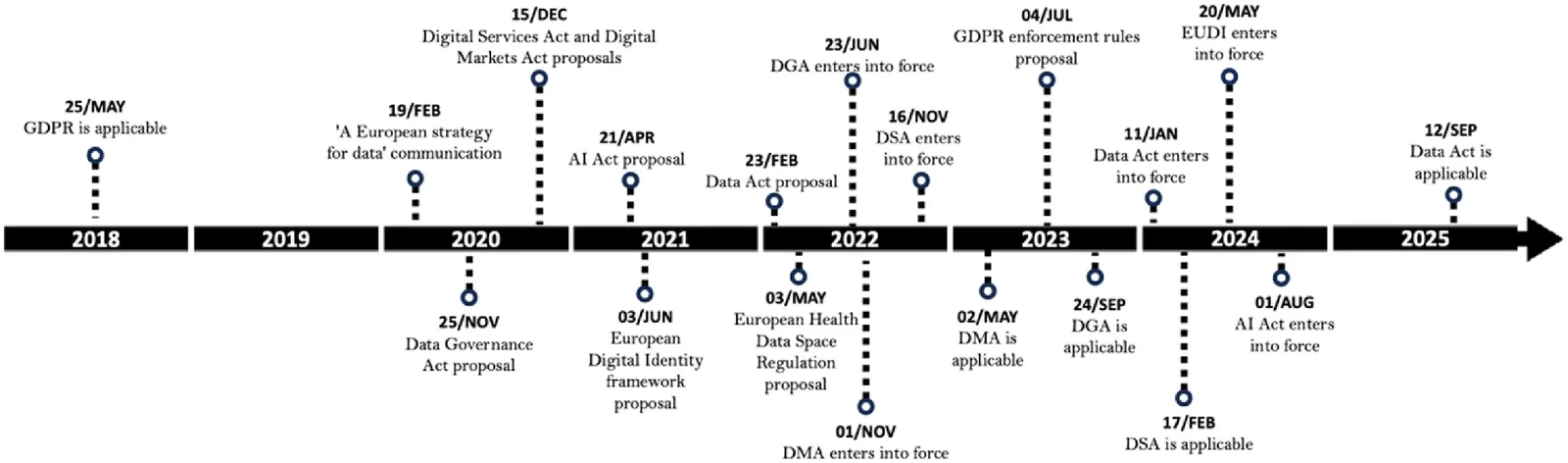
Timeline of European data and digital services regulations in the wake of the European strategy for data.

**European Digital Identity** The EUDI Framework builds on the electronic Identification, Authentication and Trust Services Regulation (eIDAS) [78]. The latter establishes a single, interoperable framework for electronic identification and trust services to enable safer electronic interactions between businesses, while making them faster and more efficient across the EU. Furthermore, trust service providers that comply with eIDAS requirements can also use their services as evidence in legal proceedings. Examples of trust services are electronic signatures, electronic seals and timestamps, website authentication certificates, or electronic registered delivery services. EUDI introduces a series of new requirements in this law, in particular including the creation of a European Digital Identity Wallet, which should be certified by certification bodies accredited by data protection supervisory authorities. Recently, the EC also adopted five implementing regulations related to *i)* rules for the integrity and core functionalities of EUDI Wallets [79], *ii)* rules on the protocols and interfaces of EUDI Wallets solutions [80], *iii)* rules on person identification data and electronic attestations of attributes of EUDI Wallets [81],

*iv)* reference standards, specifications and procedures for a certification framework for EUDI Wallets [82], and *v)* obligations for notifications to the Commission concerning the EUDI Wallet ecosystem [83]. By default, Wallet providers must remain blind to the details of a user’s Wallet-related transactions, however, an exception arises – where personal data is essential for delivering a Wallet-related service, the user’s explicit consent is required and should be granted for each specific instance. Additionally, EU countries are tasked with creating mechanisms at national level to enable qualified trust service providers in verifying “qualified electronic attestation of attributes”, i.e., official documents such as, e.g., social security documents or driving licenses. As such, these providers must be equipped to validate the authenticity of these attributes through authentic sources, but solely with the informed consent of the individual to whom the attestation pertains.

**Data Governance Act** The DGA [66] is one of the key pillars of the *strategy for data* to create a single European data space. Its primary aim is to foster the exchange of data across sectors within the EU, while embedding “transparency” requirements for data users. It sets forth conditions for the reuse of specific categories of public sector data and introduces a new entity – the single information point provider – to facilitate access between data users and this particular data domain. Beyond this, it legislates the market for data intermediation service^3^ providers and data altruism^4^ organizations, paving the way for data subjects and data holders to share their information with these entities, in the latter’s case for the “public good”. Regarding consent, none of these new services can be overlooked. When contemplating the reuse of data pertaining to data subjects, public sector bodies must enable the reuse of data on the basis of consent, ensuring that data flows only with the deliberate agreement of its subjects. To this end, they are entrusted with guiding potential reusers in seeking such consent, providing pathways through technical mechanisms to channel these requests for reuse, as well as with informing data subjects about the possibility to refuse consent (Recital 15 [66]). Furthermore, data intermediation service providers, when offering their services to data subjects, must provide avenues for individuals to wield their rights, including “giving and withdrawing their consent to data processing” (Recital 30 [66]). Additionally, data intermediation service providers that operate as data cooperatives^5^ aim to “strengthen the position of individuals in making informed choices before consenting to data use, influencing the terms and conditions of data user organizations attached to data use in a manner that gives better choices to the individual members of the group or potentially finding solutions to conflicting positions of individual members of a group on how data can be used where such data relates to several data subjects within that group” (Recital 31 [66]). As for data altruism activities, to cultivate trust in the process of granting and withdrawing consent with clarity and ease, a European data altruism consent form will be developed by the European Commission, with the support of the European Data Innovation Board and the European Data Protection Board. Especially in the realms of scientific research and statistical use of data, this form should serve as a bridge between legal terms and a user-friendly experience for altruistic data sharing (Recital 52 [66]). Nonetheless, the latest practical guide related to the DGA, published by the Commission on September 24th, 2024, does not provide any updates on when the form will be available [84], and flags are already being raised regarding the economic viability of these new services [73]. There is, however, a push for the use of standardized semantic specifications to design these forms, while maintaining the potential for personalization and contextualization [85,86]. Currently, there are at most twenty-four data intermediation services providers^6^ and two data altruism organizations^7^ registered by the Commission, while the DGA has been applicable since September 24th, 2023, and data intermediation services providers need to comply with their obligations by September 24th, 2025. When it comes to data altruism, there are ambiguities in its definition and application [87]—it focuses on consent, although consent is only one of many applicable legal grounds for the processing of personal data, and the altruistic reuse of non-personal data seems to be implicitly overlooked. Not only that, the interpretation of broad consent or how to assess compatible reuses of data still lacks clarity, while the notion of data altruism activity does not provide enough detailed information, in particular in relation to the allocation of responsibilities and the protection of individuals when it comes to their data rights. Furthermore, the Commission has abandoned the idea of having a rulebook for data altruism^8^, which was set out on DGA’s Article 22 [66] and was supposed to provide additional information related to the obligations set out for these organizations. As for data intermediation services, several issues have also been pointed out. Besides the description of potentially demanding conditions for providing such services, described in DGA’s Chapter III [66], the scope of activities and business models of intermediaries requires clarification [87,73]. The interplay of DGA’s rules with other branches of law, e.g., cybersecurity, or price regulations, must be internally unbundled by organizations seeking to supply intermediation activities, with clear guidance from the Commission and its supra-national authorities still lacking [72].

**Data Act** The Data Act [69] constitutes a cross-sectoral legislative framework and a central pillar of the European data strategy, designed to establish harmonized principles governing equitable access to and responsible use of data. It covers both personal and non-personal data, while laying down requirements on *i)* making data generated through connected products and related services accessible to the user, *ii)* making data from data holders available to data recipients and *iii)* to public sector bodies and authorities when needed for public interest tasks, *iv)* ensuring fair terms for data sharing contracts between data holders and small and medium-sized companies or not-for-profit research organizations, *v)* enabling the switch between data processing services, *vi)* establishing safeguards to protect data holders against unlawful data transfer outside the EU, and *vii)* developing interoperability standards for data to be accessed, transferred and used. Moreover, it reviews certain aspects of the 1996’s directive “on the legal protection of databases” [88], to make data generated or acquired from Internet of Things (IoT) devices accessible for access and reuse. While the Data Act is primarily concerned with non-personal data, in circumstances where users of connected products and related services qualify as data subjects, the rights conferred by the Act operate in addition to those established under the GDPR [15]. In fact, a central challenge for the Act lies in its interaction with the EU’s personal data protection framework, particularly in contexts where organizations process datasets comprising both personal and non-personal data, as this distinction is paramount to assess the scope of application of the GDPR [89]. Additionally, the rights of access and data portability set forth in GDPR’s Articles 15 and 20 [15] are further complemented by this Act by allowing users to “access all data generated by the use of a connected product, whether personal or non-personal” [69]. Accordingly, when wishing to process personal data, a GDPR legal ground such as consent needs to be in place. The influence of consent within the framework of Data Act requirements extends far beyond this point—even when data sharing agreements are automatically executed, there must be tooling in place to interrupt and terminate them with the mutual consent of the involved parties (Recital 104 [69]). Furthermore, ensuring coherence with the requirements of the DGA [66], DMA [68], and the AI Act [21], as well as with adjacent branches of law—including contractual obligations, competition law, and the protection of trade secrets—presents further potential sources of friction.

**Artificial Intelligence Act** The AI Act [21] establishes a risk-based approach to guide the governance of artificial intelligence across the EU, assigning responsibilities to providers and users according to the weight of the risks their AI systems may carry. At its sternest, the Act draws a clear boundary against high-risk applications that endanger human safety: systems designed to manipulate, to score individuals socially, to remotely identify them, or to sway the will of voters are categorically forbidden. Yet, the Act is not meant to stifle innovation—it allows general-purpose AI to flourish, provided that it safeguards the fundamental rights of EU citizens and upholds transparency through practices such as AI impact assessments. Exemptions for research endeavors and for components shared under open-source licenses are also granted. Regarding the role of consent, the Act extends its scope beyond its usage to justify personal data processing: *“informed consent”* from subjects is also required for the testing of an AI system, and its intended purpose, in real-world conditions, and following the GDPR counterpart, it may be revoked at any time without providing any justification.

**European Health Data Space Regulation** The EHDS Regulation [71] is the first legislative initiative in the EU aimed at establishing a common European data space. Its objective is to empower individuals by granting them improved access to their electronic personal health data, both at the national and European levels. In parallel, the Regulation seeks to establish governance mechanisms for primary and secondary use of electronic health data, and foster the development of a unified market for electronic health record (EHR) systems, wellness applications, and medical devices. In addition to extending the rights of access, rectification, and data portability established in Articles 15, 16, and 20 of the GDPR [15], this Regulation grants data subjects, specifically with regard to personal electronic health data, the following rights: *i)* to insert information in their own EHR, *ii)* to restrict access, *iii)* to obtain information on accessing data, *iv)* to opt out in primary use, and *v)* to lodge a complaint with a digital health authority. Furthermore, by 26 March 2027, the EC will launch, through implementing acts, technical specifications for the priority categories of personal electronic health data, namely *i)* patient summaries, *ii)* electronic prescriptions, *iii)* electronic dispensations, *iv)* medical imaging studies and related imaging reports, *v)* medical test results, and *vi)* discharge reports. These specifications will define the European electronic health record exchange format, which must be commonly adopted in the EU, machine-readable, and enable the transmission of personal electronic health data across different software applications, devices, and healthcare providers. By that same deadline, the Commission will also give shape to a central interoperability platform for digital health. This infrastructure will provide services to enable the exchange of personal electronic health data among the Member States’ national contact points for digital health. Moreover, the EHDS Regulation provides a set of measures to ensure a consistent, and trustworthy secondary use of health data. It establishes the minimum categories of electronic health data that data holders must make available for secondary use, such as electronic health data from EHRs or data from wellness applications, as well as the purposes for which it can be processed, e.g., improvement of the delivery of care, scientific research related to health, and policymaking and regulatory activities. Nonetheless, data subjects have the right to opt out of secondary use at any time without providing any justification. Be it for primary care or for the secondary use of data, every activity including the processing electronic health data must rest upon a GDPR lawful ground (Recitals 15 and 52 [71]). As such, within this framework, consent emerges once more as a pivotal subject matter, particularly in light of the sensitivity that health data embodies.

The misuse and overuse of consent, as a generic mechanism to exchange personal data on the Web, is a well-known fact by now. Not only that, the transactional speed at which it is happening also serves as a confirmation that it cannot be considered valid consent. With the key role that consent was given within the EU’s data and technology regulatory landscape, one might wonder whether the issues that consent has been facing as a legal basis for personal data processing are not being seriously debated. It seems that we are already past what consent can make possible for both individuals and companies, while we demand more to fulfil the common European data spaces. In this context, we explore the advantages and disadvantages of going beyond consent, exploring the myriad of other lawful grounds that allow the processing of personal data.

## 3 Beyond Consent

### 3.1 The lawful six

Consent is only one of the lawful grounds prescribed by the GDPR for data controllers to process personal data of EU citizens. Beyond the oft-cited ground of consent, Article 6 [15] provides a framework of alternative legal bases, each carrying its own rationale and applicability. *Contractual necessity* enables data processing when it is necessary for fulfilling contractual agreements in which the data subject is a party. *Legal obligation*, by contrast, reflects the authority of law itself, compelling controllers to comply with its provisions. *Legitimate interest* occupies a more nuanced position, inviting a balancing of organizational aims against the rights and freedoms of individuals. The ground of *public interest* allows processing where the welfare of society outweighs private considerations, including the activities of official authorities. Finally, *vital interest*, more rarely invoked, provides a safeguard in moments of urgency for the protection of life or safety of data subjects.

The determination of an appropriate legal basis for processing under the GDPR is inherently dependent on the specific purpose of the processing activity, as well as the nature of the data involved and other contextual factors such as the relationship between the controller and the data subject. The choice of legal ground must therefore be carefully aligned with the underlying rationale for processing and the risks associated with the data at issue. Importantly, when these conditions evolve—for example, if the purposes of processing change—it becomes necessary to re-evaluate the chosen legal basis and determine whether a new one must be established to ensure continued compliance. Table 2 presents a comparative analysis of the previously identified lawful bases, evaluating them across several dimensions of practical relevance. Specifically, it considers their potential for automation, that is, the extent to which a given legal basis may enable organizations to automate or semi-automate access to personal data. It further examines the degree of involvement required from the data subject, highlighting variations in the level of individual intervention. Finally, the table assesses scalability, understood as the ease with which a legal basis can be consistently applied at scale across multiple services and jurisdictions. These dimensions are particularly relevant for organizations seeking to streamline compliance processes while ensuring that individual rights remain adequately protected. Furthermore, this analysis applies exclusively to categories of personal data that do not fall within the GDPR’s definition of special category data, for which additional safeguards and legal conditions are required.

**Table 2:** Comparison of GDPR legal grounds in terms of automation, data subject interaction, and scalability.

Legal basis	Automation potential	Data subject interaction	Scalability
**Consent**	Low	High	Low
**Contract**	High	Medium-Low	High
**Legal obligation**	High	Low	High
**Legitimate interest**	Semi	Medium	Medium
**Public interest**	Low	Low	Low
**Vital interest**	Semi	None	Low

From the perspective of *automation potential*, contract and legal obligation emerge as the most amenable to deterministic, rule-based implementations. Contractual processing can often be operationalized in a straightforward manner, as access decisions follow directly from the predefined terms of the agreement between the parties. Similarly, legal obligation lends itself to automation where obligations are clearly codified, though complexity may arise when overlapping or conflicting legal regimes demand context-specific interpretation. Legitimate interest and vital interest, by contrast, allow only partial automation: both require human oversight, either through a contextual balancing test in the case of legitimate interest, or through predefined emergency scenarios in the case of vital interest. Consent and public interest remain the least conducive to automation. Consent would require highly granular data subject preferences to account for the diverse range of purposes, data types, and data controllers involved, while public interest processing is typically confined to specific authorities and thus not easily generalized across contexts.

The dimension of *data subject interaction* further illustrates the varying operational implications of each legal basis. Consent requires the highest level of engagement, encompassing opt-in mechanisms, ongoing policy management to manage one’s preferences, and the possibility of withdrawal. Legitimate interest involves a more limited form of interaction, namely the right of the data subject to object to processing. Contractual processing entails interaction at the point of sign-up or acceptance, after which further data subject involvement is minimal. Legal obligation and public interest grounds typically preclude any opt-out, as compliance with statutory or societal requirements takes precedence over individual choice. Vital interest, by definition, applies in circumstances where the data subject is unable to act, thereby eliminating direct interaction altogether.

In terms of *scalability*, contract and legal obligation again stand out. Standardized contractual terms make it possible to extend the same legal ground across large numbers of users, while legal obligations enable the construction of compliance pipelines around well-defined statutory requirements. Legitimate interest is somewhat more limited, but still more scalable than consent, since it avoids the administrative overhead of user-by-user consent collection. While the balancing test introduces interpretive complexity, standardized assessment tools could partially mitigate this burden. By contrast, consent suffers from inherent scalability challenges, mostly related to its informed and specific character. Public interest and vital interest grounds are even more narrowly circumscribed: the former applies predominantly to specific public bodies, while the latter is invoked only in exceptional, often life-threatening situations.

In conclusion, the analysis suggests that the contractual legal ground offers significant potential for both automation and scalability, as its reliance on predefined agreements allows organizations to implement standardized agreements as the basis for rule-based personal data processing activities, while contract law does not forbid such arrangements, given that the legal limits when processing special data are strictly respected. Nettesheim shares this view by stating that “that effective data protection in contractual relationships can and must be realized above all through an appropriate design of the contractual relationships” [90]. Accordingly, contractual necessity should only be used as a processing ground where appropriate, i.e., for the provision of the requested service, and not to expand data processing for additional purposes that are not covered by the contract, e,g., for the sole benefit of the data controller’s commercial interests [91], or for the establishment of unilaterally imposed agreements containing unfair terms for service consumers [92]. Contract is also a recognized and operative legal basis in several EU laws beyond just the GDPR, namely under the Data Act [69], to use data generated by connected products. Similarly, the legal obligation ground demonstrates strong suitability for automated and scalable processing, though its applicability remains confined to contexts where regulatory compliance explicitly requires the handling of personal data. Hence, both lawful grounds should be used where consent should not be due to an inherent power imbalance between controller and subject, for instance, when employers are processing the personal data of their employees [93]. Legitimate interest, by contrast, with its broad and not purpose-specific scope [94], should be considered to enable exchanges that can create value for both controllers and data subjects, even if they override the latter’s interests at times. Accordingly, the performance of balancing exercises in every distinct context is important to avoid misuse and protect the interests of data subjects [95]. Overall, broadening the range of lawful bases beyond consent enables data controllers to maintain compliance with the GDPR, while simultaneously alleviating individuals from the considerable scalability and comprehensibility challenges that consent would pose if it were required for all personal data processing activities encountered in daily life.

### 3.2 Jurisdictional flavors

While the EU’s GDPR establishes a comprehensive framework with six primary legal bases, other regions either adopt similar structures or deviate by introducing unique grounds or limiting their scope of applicability. The comparative overview in Table 3 illustrates both the convergence and divergence of legal bases for personal data processing across major jurisdictions—previously described in Section 2.1.

**Table 3:** Legal basis from the several data protection regulations described in Section 2.1, including the EU's GDPR for comparison. X is used to identify the legal bases present in each regulatory text. O is used to differentiate opt-out consent from its opt-in counterpart—all US state laws only include opt-out consent. In the case of India, (X) marks legal bases that are covered by the “Legitimate uses” legal basis present in the DPDP Act. Pakistan's law is represented in X as it is still a draft and, as such, subject to change.

Legal bases	EU	IN	CN	US	ID	PK	NG	BR	BD	RU	MX
**Consent**	X	X	X	O	X	X	X	X		X	O
**Contract**	X		X		X	X	X	X		X	
**Legal obligation**	X	(X)	X		X	X	X	X		X	
**Vital interest**	X	(X)	X		X	X	X	X		X	
**Public interest**	X	(X)	X		X		X				
**Legitimate interest**	X				X	X	X	X		X	
**Legitimate uses**		X									
**Legally disclosed**			X							X	
**Disclosed by DS**			X							X	
**Required by law**			X			X				X	
**Public administration**								X			
**Research**								X		X	
**Rights exercise**								X			
**Credit protection**								X			
**Media and journalism**										X	

As previously established, in most jurisdictions, *consent* serves as a foundational basis, whereas in the US state laws and Mexico, it operates primarily as an opt-out mechanism with certain opt-in requirements for sensitive data. Contract and legal obligation also exhibit substantial alignment across several jurisdictions. In the EU, Brazil, Russia, Indonesia, Pakistan, Nigeria, and China, *contracts* provide a legitimate basis where the processing is necessary for contractual performance. *Legal obligation*, similarly, is a consistent ground across these frameworks, except for China, though its interpretation may differ. Beyond legal obligation with court orders or statutory duties, in China, Pakistan and Russia, personal data can be processed if *required by other laws or administrative regulations*.

Certain jurisdictions expand the list of recognized grounds with context-specific provisions absent from the GDPR. India’s DPDP Act [23] introduces *legitimate uses*, encompassing situations such as voluntary data provision, medical emergencies, public health, employment, or legal obligations, leaving out contractual necessity or legitimate interests. China explicitly recognizes personal data *disclosed by the individual* or made *legally public*, with the Russia’s DPA law [56] providing akin bases. Brazil introduces additional categories including *research, exercise of rights* in judicial, administrative or arbitration proceedings, *credit protection*, and *processing by public administration* for policy execution. Russia similarly codifies provisions for *media and journalism*, as well as research. These specific, context-driven legal bases reflect a pragmatic accommodation of socio-economic and institutional needs within national data protection regimes. At the same time, *public interest* and *vital interest* remain unevenly adopted. The EU, China, Indonesia, and Nigeria acknowledge these categories, particularly for tasks carried out by public authorities or in emergency situations, but their absence in other jurisdictions is either folded into more specific categories, e.g., legitimate uses in India or research in Brazil and Russia, or addressed through sector-specific rules rather than general data protection law.

A striking divergence emerges in the United States and Mexico, where opting out is the norm. Opt-in is reserved for the handling of sensitive data, including child data, in the California, Colorado, Connecticut, Oregon, Texas, and Virginia state laws, while Nevada’s opt-in consent mechanism concerns only health data, and the Mexican law goes one step further by mandating written consent for all sensitive personal data. This structure underscores a consumer protection orientation rather than a legal basis framework in the European sense.

Taken together, these findings reveal a fragmented but partially convergent global landscape. While consent, contract, and legal obligation form common denominators across many systems, several jurisdictions supplement these with additional, context-specific grounds. Such variations reflect differing legal traditions, regulatory priorities, and socio-political contexts, ultimately shaping how data controllers and subjects experience the balance between flexibility, compliance, and protection. Nonetheless, the contractual legal basis warrants particular emphasis, as it not only enables greater automation and scalability compared to consent, but also provides a lawful foundation for processing personal data across a wide range of jurisdictions, thereby offering long-term benefits for both controllers and data subjects. In the following section, we delve into the concept of *evolvable trust* —a scalable technolegal alternative that explores GDPR’s legal bases without overreliance on consent as the sole lawful ground, while simultaneously establishing economic incentives for the exchange of data, goods, and services among the parties involved.

## 4 Trust as a Temporal Relationship

### 4.1 Defining trust

Despite abundant mentions of the word “trust”, we have so far not defined it, as such definitions tend to stray into philosophy. To mitigate this risk, we present an operational definition for the purposes of this article:

**Trust** is a relationship between individual people or groups of individuals, the quality of which evolves over time, serving as a channel that supports the exchange of data, services, and/or goods.

From a legal perspective, this definition is not intended to introduce “trust” as an autonomous legal construct or lawful basis for processing. Rather, trust is understood here as a relational and temporal phenomenon that underlies and shapes the application of existing legal frameworks. Evolvable trust therefore operates as a governance-oriented concept that informs how legal bases are interpreted and operationalized over time. In this sense, it does not replace legal doctrine, but provides a structuring lens through which its dynamic application can be better understood.

Furthermore, by defining trust as an interpersonal relationship, we explicitly exclude relationships between people and devices or software; the idea being that people fundamentally cannot bestow trust upon technology, for technology can impossibly experience or reciprocate trust. As such, in our model, people trust companies, even if such companies are represented by software. The role of technology is not that of a party in the trust relationship, but rather to support such relationships at scale.

Current technologies are not well equipped to support the inevitable evolution that characterizes the dynamics of human trust relationships. As such, the potential for improvements on each side of the relationship is substantial. We compare the current, limited model with a model that better reflects the complexity of the real world.

### 4.2 Single-instance trust establishment

In current practical interpretations of the GDPR, data-exchange procedures are, in the majority of cases, structured on the basis of a single-instance temporal model, in which the operative decisions concerning the terms of the relationship between the parties are concentrated at the initial point of interaction. The prevailing configuration adopted by data controllers may accordingly be characterized as a single-Instance trust establishment model, in which the initial point of interaction with the data subject is treated as the sole occasion on which the guarantees and permissions applicable to the subsequent relationship are to be obtained. Fig. 5 refers to this moment as a Brief Expression of Trust (BEOT), providing a representation of a timeline within the parameters of this model, on the basis of an example in which an individual data subject establishes a relationship with a provider of a music-streaming service. The initial visit to the website of the service provider corresponds to the point at which the data subject is required to make all decisions concerning the terms of the relationship, notwithstanding the fact that the relationship in question has not yet commenced. The configuration presents the operational advantage that any subsequent act of data sharing is covered by the single prior agreement; it presents the corresponding limitation that the agreement must be drafted more broadly than would be necessary for any individual data exchange occurring under it.

**Fig. 5:**
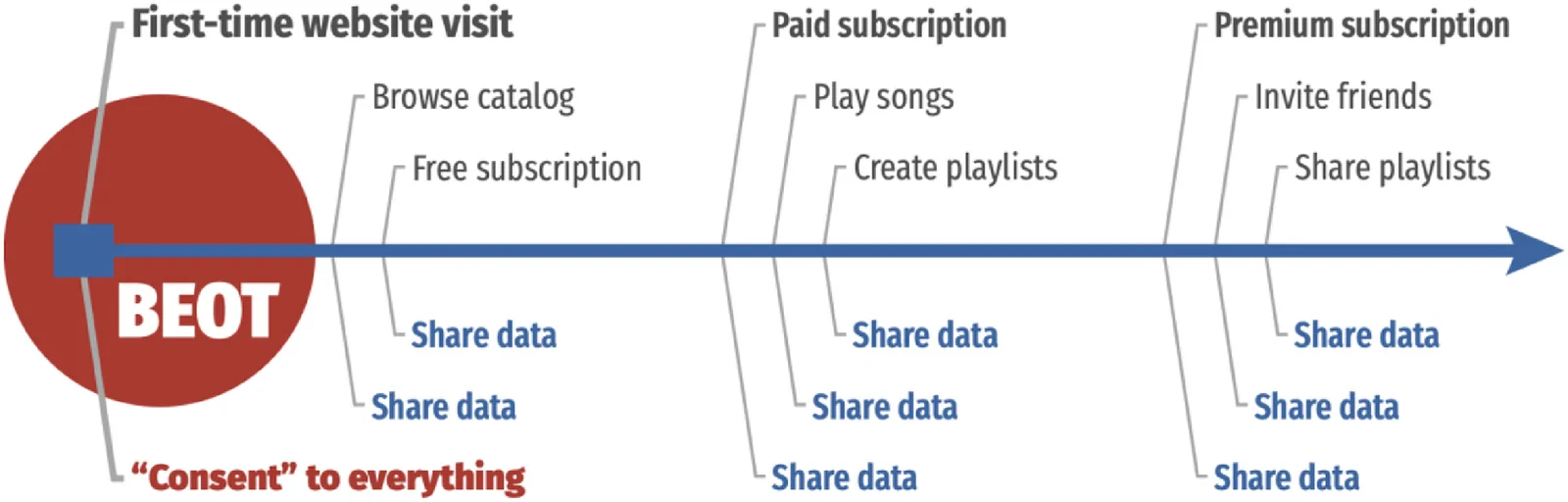
The dominant timeline for digital trust relationships starts with the Brief Expression Of Trust (BEOT), at which point a data subject is asked to agree to an exhausting list of demands, in stark contrast to the absence of a factual trust relationship that would normally serve as collateral. Informed decision or negotiation is impossible as the relationship itself has not yet commenced, leaving no opportunities to assess trustworthiness. All needed and unneeded conditions are established beforehand, as the consent dialog model disregards the evolutive character of real-world relationships.

The uncertainty stems from the presumption that anything left uncreated at the start of the joint timeline with a data subject might never come into existence. Assuming this model of the data universe, a controller’s only rational behavior consists of seizing the initial moment of the BEOT to demand a data subject’s unconditional and non-negotiable consent for all possible data usages, thereby already anticipating any possible timeline developments for themselves or their current and future partners. As a result, the data subject is asked to settle all of the trust that could possibly be required for the entire duration of the relationship before the relationship takes place—by essentially agreeing to its absence.

Unfortunately, the cookie mechanism limits both parties, as the ability to evolve is crucial to B2C relationships. We recall that a lack of trust incurs a significant cost for people and companies. From a legal angle, it is doubtful whether the threshold of informed consent is achieved. Nonetheless, the agreement texts themselves likely provide some degree of protection due to their sheer length, which could make litigation prohibitively expensive. At the same time, they paint a grim picture of the future, especially for businesses that aim to establish a genuine relationship for mutual benefit—which not coincidentally forms the cornerstone of economics. From a doctrinal standpoint, the BEOT model reflects a convergence of legal and practical constraints, including the static nature of contracts of adhesion and the transactional interpretation of lawful bases such as consent. While these mechanisms provide formal legal certainty, they struggle to capture the inherently temporal and relational nature of trust. Companies cannot leverage trust as a key differentiator with a static interaction model that imposes fixed boundaries at the start. Evolvable trust can thus be understood as a response to this limitation, seeking to complement existing legal structures with mechanisms that allow for continuous recalibration rather than one-off legitimization.

### 4.3 Evolvable trust

Evolvable trust should be understood as a framework that operates across, rather than within, established legal categories. It is neither reducible to a contractual construct nor confined to a specific compliance mechanism. Instead, it provides a governance layer through which different legal bases—such as consent, contractual necessity, or legitimate interest—can be dynamically instantiated and combined in a manner that reflects the evolving state of the relationship between the parties, enabling a continued relational support via smaller data transactions.

In a model of evolvable trust, the qualities of trust in the model need to reflect those of the real-world at any given point in time. Specifically, the following conditions need to hold:

• There is no presumption of trust at the start of the relationship.
• The quality of trust can evolve in a positive or negative direction at any point in time.
• Every act of exchange is contextualized with specific policies and provenance that make the understanding of trust at the moment explicit.

These conditions reflect a shift from static legal qualification towards continuous legal contextualization, in which the applicable legal basis and associated safeguards are determined in relation to the specific moment of exchange. As an illustration of these conditions, the timeline in Fig. 6 reimagines the interaction of Fig. 5 in which a person establishes a new relationship with a streaming music provider. The relationship starts when the person visits the website, at which point there exists no notion of trust between both parties. Through interaction with the website, the person gains confidence that the company can provide value to them, yet no exchange has taken place yet.

**Fig. 6:**
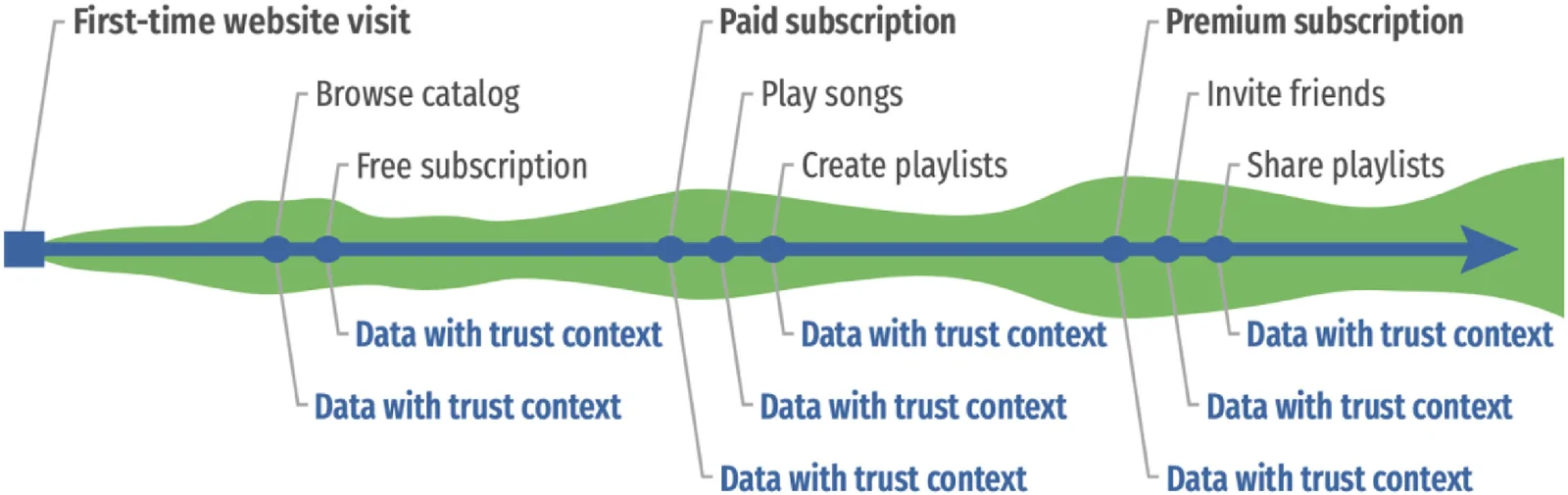
In an evolvable trust model, no decision or exchange happens at the first moment of interaction; rather, the relationship is allowed and encourage to grow. Every act of exchanging data is encapsulated in a trust context pertaining to this specific data point and exchange, reflecting the status of the relationship at that moment in time. This model aligns with the dynamics of real-world relationships, notably due do its elimination of widely scoped prior agreements regarding abstract future interactions that cannot be meaningfully decided yet.

By browsing specific parts of the music catalog, the person discloses the first piece of personal data, namely their interest in certain artists and genres. This specific act of exchange in isolation could be argued from the perspective of legitimate interest, since disclosing a preference for a certain genre is a necessary precondition to display it. As such, the data point “this person prefers 80s rock music” is exchanged with the context that the person considers the usage of this data for the selection of songs during the active browsing session to be of legitimate interest to the person and the company. At this point, the person can back out of the relationship with the understanding that their exchange of data will not impact them any further, given the specific scoping in time and purpose. The company has achieved its goal of demonstrating they provide a service relevant to the person’s musical tastes.

The company subsequently offers a free subscription, which can be activated by creating an account through a combination of email address and password. These individual data points constitute personal information, which can similarly be exchanged with a specifically scoped legal basis.

This mechanism is invoked repeatedly to support the evolution of the relationship at every step. When a stronger engagement is required, such as when the person decides to purchase a paid subscription, explicit consent will be asked for the credit card information to be used for this specific transaction. At every point in the relationship, each exchanged data point is accompanied by the minimal trust context required to achieve every step. This does not imply the creation of a new legal basis, but rather illustrates how existing bases can be applied in a granular and context-sensitive manner when supported by appropriate technical and organizational safeguards for data exchange. A potential risk of this incremental model lies in the gradual expansion of the scope of personal data collected over time as the relationship deepens. While such expansion is, to an extent, an intended feature—reflecting the increasing range or quality of services requested—it must not result in an unchecked accumulation of personal data. To mitigate this risk, evolvable trust requires that each incremental data exchange remains independently justified according to the principles of necessity, proportionality, and purpose limitation. In contrast to the BEOT model, where excessive data collection is frontloaded “just in case”, the proposed approach enforces a “just-in-time” logic in which data is only requested when it becomes strictly relevant to a newly established context of use. The advantage of this approach is that the company never requires a level of trust or agreement beyond what is strictly necessary, and that all the information to make an informed decision is available to the person at the point where they have to decide. The disadvantage is, compared to the single action pattern of the BEOT, the large number of individual decision points during the course of the relationship timeline. However, their vastly increased volume is compensated by a significant narrowing in scope for each point, opening up the possibility of assistance or even automation.

### 4.4 Assisted or automated trust decisions

The development of assisted and automated trust systems requires a rethinking of how individuals, organizations, and machines interact in the exchange of data, services, and goods. Evolvable trust cannot be sustainably operationalized under the current paradigm of data usage management if, instead of a BEOT, millions of decisions have to be taken by companies and people alike. To move beyond this impasse, a higher level of abstraction is necessary for the representation of data usage conditions. Rather than requiring individuals to engage with the minutiae of every data transaction, meta-policy languages and reusable policy catalogs can provide scalable mechanisms for defining, deriving, and instantiating policies that align with individual preferences, legal requirements, and organizational needs. These policy-based frameworks would allow for contextual instantiation at the moment of decision-making, enabling the selection of appropriate legal bases—including but not limited to consent—under the GDPR and other regulatory systems. This does not preclude consent playing a role in “evolvable trust” ecosystems, however its applicability should be constrained to one-off transactions where it remains appropriate. In this context, existing standards such as the W3C’s Open Digital Rights Language (ODRL) [96] provide a machine-readable framework for expressing permissions, prohibitions, and obligations associated with data use, while vocabularies such as the Data Privacy Vocabulary (DPV) [97] enable the semantic representation of legal concepts including purposes, legal bases, and processing operations. Together, these technologies support the formalization of data usage policies in a manner that is both legally meaningful and technically enforceable. These policies can then be encapsulated within so-called trust envelopes—structured artifacts that bind data together with its associated usage conditions, legal context, and provenance metadata [98]. By maintaining this binding across the data exchange lifecycle, trust envelopes ensure that the semantics governing data use remain attached to the data itself, enabling consistent interpretation and enforcement across different systems and actors. Based on these technologies, both assistance and automation mechanisms could support individuals and companies in the evolution of their relationship.

Furthermore, such mechanisms require dynamic negotiation protocols if they are to support the exchange of a data point with a trust context that includes *specific* data usage conditions. Negotiation protocols, expressed in standardized and machine-readable formats, enable individual preferences and controller requirements to be reconciled efficiently, independently of the legal ground that justifies such a transaction. To ensure that both individuals and companies can meaningfully engage with these negotiations, personalized dialogs are indispensable. Under consent, this means tailoring information to support informed choices, whereas in the context of legitimate interest, dialogs may highlight the right to object. Personalized interfaces, when adaptive and responsive to individuals’ distinct wishes for control, enhance autonomy and ensure that reliance on any given legal basis is both valid and transparent. The delegation of control further extends this approach by enabling individuals to entrust decision-making to other individuals or automated agents. For example, consent managers or intelligent policy agents can enforce predefined user preferences while adapting to evolving contexts. India’s *consent manager* [23] and US-based *authorized agents* [47,49,50] represent regulatory efforts that incentivize the use of such technologies.

Complementing these measures, auditing mechanisms ensure that assisted and automated processes remain accountable over time. Concurrently, automation should not be understood as a substitute for institutional oversight. We fully acknowledge that the power asymmetries inherent to the data economy cannot be resolved through technical means alone. Evolvable trust is therefore conceived as a complement to, rather than a replacement for, existing supervisory frameworks. By encoding legal constraints into the technical data flow, the proposed approach introduces a form of compliance-by-design that enables continuous, verifiable enforcement of legal requirements. This, in turn, provides supervisory authorities with a transparent and auditable trail of data processing activities, strengthening their ability to monitor, investigate, and intervene where necessary. As such, integrated auditing functions strengthen both ex-ante and ex-post accountability, reassuring individuals that their data is handled as promised while equipping organizations with demonstrable evidence of compliance.

In this context, compliance and provenance checking emerge as foundational components of assisted and automated trust systems. Each data transaction is not only evaluated against applicable legal constraints prior to execution but is also accompanied by verifiable metadata describing its origin, related rights’ holders, and conditions for usage. Trust envelopes provide a concrete mechanism for structuring this metadata, combining, as previously mentioned, technologies such as ODRL or DPV, for policy expression, with standardized metadata vocabularies such as DCAT (Data Catalog Vocabulary) [99] and PROV-O (Provenance Ontology) [17] to leverage dataset descriptions and their processing provenance in a structured and interoperable manner. Provenance-aware systems enable both individuals and supervisory authorities to reconstruct the lifecycle of a data point across time and organizational boundaries, ensuring that downstream processing remains consistent with the conditions under which the data was initially disclosed. This continuous validation of data flows operationalizes accountability beyond static documentation, embedding compliance directly into the infrastructure of data exchange.

Finally, the question of secondary data reuse highlights the need to balance innovation with sustained accountability and stakeholder alignment. While evolvable trust enables the incremental expansion of data use, any transition from primary to secondary purposes must be explicitly governed through renewed contextualization, including reassessment of legal bases, and, where necessary, user involvement. At the same time, secondary reuse creates opportunities for shared value generation, particularly when individuals are meaningfully included in the decision-making process or benefit from the outcomes of such reuse.

Taken together, these areas illustrate that assisted and automated trust decisions must operate across multiple layers of governance, communication, and oversight. Trust envelopes can act as a unifying construct that bridges these layers by coupling data with enforceable policies, verifiable provenance, and interoperable semantics, thereby enabling the practical realization of evolvable trust across heterogeneous systems. Their integration provides not only a scalable and interoperable infrastructure for data exchange but also a pathway to establishing evolvable trust relationships that endure over time.

## 5 Conclusions

The prevailing reliance on consent dialogs may be considered to reflect a limited degree of coordination among the technological, legal, and economic disciplines. On the one hand, contemporary technologies are frequently characterized as capable of supporting profit-maximization strategies premised on the extensive collection of personal data through the full range of legally available and adjacent means. On the other hand, the same technologies are frequently characterized as insufficient with respect to the capture of the relational complexities that are advanced as justification for the processing of the corresponding volumes of personal data. The characterization of significant instances of distrust as inevitable may be considered to reflect, rather than to address, the underlying limitations with respect to competitive positioning.

Trust is not a zero-sum construct. On this basis, it may be understood not as a matter of the allocation of control among the parties to a relationship, but rather as a matter of the exercise of self-determination within such relationships, with associated benefits for all parties concerned. Substantive autonomy may be considered to arise under conditions in which data subjects retain the capacity to exert ongoing influence on the manner in which their personal data contributes to the evolution of a given relationship, without being required to select between the abstract configurations of complete confidentiality and complete disclosure. In contrast to a consent model of a single-instance and binary character, trust may be characterized as a personalized equilibrium among competing considerations, including confidentiality and availability, conciseness and transparency, and accountability and privacy, each of which may be subject to adjustment on a per-relationship basis over time. Personalization implemented in a responsible manner may contribute to the resolution of situations characterized by asymmetric distribution of benefits, by aligning the configuration of control mechanisms with the interests of the individual data subject, in preference to the application of uniform rules that may prove suboptimal for both data subjects and commercial undertakings.

The implementation of evolvable trust relationships requires systems the design of which reduces the cost and operational effort associated with conduct aligned with the ethically preferred course of action. Data subjects are required to be in a position to delegate decisions of a nuanced character to tools operating in their interest, on the basis of a shared understanding, between human and machine actors, of the contextually appropriate parameters of such decisions. Systems of this type may be characterized as modelling trust not as a supplementary layer situated above consent, but as the broader framework within which the concepts of permission and consent are subject to evolution over time. The point of departure for the present analysis is the observation that both parties to a given relationship may derive greater value from a proportional exchange, in a manner analogous to the core principles of economic exchange. The analogy to economic exchange should not, however, be interpreted as implying the subordination of fundamental rights to market-based reasoning. The premise of the present work is, on the contrary, that fundamental rights, as codified in European data protection legislation, constitute the boundary conditions within which any exchange of personal data is required to take place. The tension identified in the preceding sections arises not from any circumstance in which economic reasoning may be understood to override such rights, but rather from operational configurations in which the practical exercise of such rights by data subjects is associated with disproportionate costs in terms of time, operational effort, or foregone utility. A binary framing may accordingly arise, in which data subjects are positioned as required to select between effective participation in digital ecosystems and the substantive exercise of the rights in question. The concept of evolvable trust may be understood as oriented towards the resolution of this tension by means of the alignment of economic incentives with the normative objectives of the applicable legal framework, such that the protection of fundamental rights is associated with the lowest available level of implementation friction, in preference to a configuration in which it operates as an impediment.

In relation to considerations of confidentiality, personal data exhibits characteristics that differ materially from those of other resources associated with the conduct of daily life, in particular the property that personal data may be reproduced at negligible marginal cost. The enhancement of the scalability of the applicable legal mechanisms is required to be predicated on the position that measures oriented towards the facilitation of well-functioning data flows, as distinct from measures oriented exclusively towards the imposition of penalties on non-compliant conduct, may contribute to the realization of proportionate benefits accruing to the data subject to whom the relevant data pertain. Notwithstanding the adverse effects on individuals, undertakings, and society associated with documented instances of inappropriate treatment of personal data, the value potential associated with personal data that remains under the control of the corresponding data subject may be considered to exceed, by a substantial margin, the value that any single commercial entity may derive from a subset of such data. Given the value potential associated with each data point, an appropriate framing of progress may be considered to consist not in the cessation of value-extraction activities in their entirety, but rather in the reallocation of the associated value to the data subjects to whom the underlying data pertain. Economic incentive structures are accordingly required to be aligned with socio-ethical considerations relating to longer-term sustainability, an alignment which entails, as a subsequent step, incorporation into the applicable legal framework. It is anticipated that an improved understanding of the trade-offs between the properties of confidentiality and availability, in combination with the automation opportunities associated with data-exchange mechanisms situated outside the framework of consent, may support the development of more effective data-flow arrangements delivering mutual benefit within the parameters established by the applicable data protection legislation.

## Ethics Statement

The authors have read and follow the ethical requirements for publication in Data in Brief and confirming that the current work does not involve human subjects, animal experiments, or any data collected from social media platforms.

## CRediT authorship contribution statement

**Beatriz Esteves:** Conceptualization, Writing – original draft, Writing – review & editing. **Ruben Verborgh:** Conceptualization, Writing – original draft, Writing – review & editing.

## Footnotes

1 Information derived from https://www.population.un.org/wpp/ (accessed on August 12th, 2024).

2 Country codes and their subdivisions are represented using the Alpha-2 code in the ISO 3166 standard, openly available at https://www.iso.org/iso-3166-country-codes.html

3 `data intermediation service’ means a service which aims to establish commercial relationships for the purposes of data sharing between an undetermined number of data subjects and data holders on the one hand and data users on the other, through technical, legal or other means [...] (Article 2.11 [66])

4 `data altruism’ means the voluntary sharing of data on the basis of the consent of data subjects to process personal data pertaining to them [...] without seeking or receiving a reward that goes beyond compensation related to the costs that they incur where they make their data available for objectives of general interest [...], such as healthcare, combating climate change, improving mobility, facilitating the development, production and dissemination of official statistics, improving the provision of public services, public policy making or scientific research purposes in the general interest (Article 2.16 [66]) 5“services of data cooperatives” means data intermediation services offered by an organizational structure constituted by data subjects, one-person undertakings or SMEs [...] (Article 2.15 [66])

5 https://www.digital-strategy.ec.europa.eu/en/policies/data-intermediary-services, accessed on August 4th, 2025.

6 https://www.digital-strategy.ec.europa.eu/en/policies/data-altruism-organisations, accessed on August 4th, 2025.

7 https://www.ec.europa.eu/info/law/better-regulation/have-your-say/initiatives/14094-Data-altruism-Rulebook-for-organisations-collecting-personal-data-in-the-general-interest_en, accessed on August 4th, 2025.

8 https://ec.europa.eu/info/law/better-regulation/have-your-say/initiatives/14094-Data-altruism-Rulebook-for-organisations-collecting-personal-data-in-the-general-interest_en, accessed on August 4th, 2025.
